# Effects of Cowpea mottle virus and Cucumber mosaic virus on six Soybean (*Glycine max *L.) cultivars

**DOI:** 10.1186/1743-422X-6-220

**Published:** 2009-12-10

**Authors:** Olawale Arogundade, Samuel O Balogun, Taiye H Aliyu

**Affiliations:** 1 Department of Crop Protection, Faculty of Agriculture, University of Ilorin, Tanke, Ilorin, Kwara State, 23401(031), Nigeria

## Abstract

The study was carried out to determine the comparative pathogenic response of six cultivars of soybean; TGx 1844-18E, TGx 1448-2E, TGx 1910-8F, TGx 1019-2EN, TGx 1910-8F and TGx 1876-4E to single and mixed infections with cowpea mottle virus and cucumber mosaic virus. The experiment was conducted in the screenhouse at the crop production pavilion, Faculty of Agriculture, University of Ilorin, Ilorin, Kwara state Nigeria. The results of the experiment revealed that all soybean cultivars were susceptible to single and mixed infection of the two viruses but to seemingly different extent. The single infection with cowpea mottle virus (CMeV), however, caused the most severe symptoms on the soybean cultivars. Cucumber mosaic virus (CMV) alone was not as severe as the CMeV. The mixed infection of CMeV and CMV did not cause higher severity than CMeV alone indicating that there was little or no synergistic effect between the two viruses on soybean.

## Introduction

Soybean, *Glycine max *(L) Merrill, is one of the oldest of cultivated leguminous Oilseed belonging to the family *Fabaceae*. It grows in tropical, subtropical, and temperate climates. It has 40 chromosomes (2n = 40) and is a self-fertilized species with less than 1% out-crossing [1]. It is an annual plant that varies in growth habit and height. It may grow prostrate, not growing higher than 20 cm, or even up to 2 meters in height [1].

Soybean is a source of high quality and inexpensive protein, which is about 40% of the seed [2]. The oil and protein contents in soybean together account for about 60% of dry soybean seed by weight, protein at 40% and oil at 20%. The remainder consists of 35% carbohydrate and about 5% ash. The oil is high in essential fatty acids, devoid of cholesterol and constitutes more than 50% of the world's edible vegetable oil in trade [3]. All these advantages notwithstanding; the crop is faced with diseases such as rust, red leaf blotch, frog-eye leafspot, bacterial pustule, bacterial blight, and soybean mosaic virus among other virus diseases, are problems to be resolved in soybean. Soybean mosaic virus (SMV) is the most frequently isolated virus of soybean, it probably occurs wherever soybean is grown, the symptoms vary according to the particular viral strain, host genotype, weather and time of infection [4]. Cowpea mild mottle virus (CCMV) has been reported on soybean from Nigeria [5,6]. Cowpea mottle virus is of localized importance on cowpea in Nigeria [7].

Cucumber mosaic virus (CMV) is worldwide in distribution. The virus causing cucumber mosaic has a wider range of hosts and attacks a greater variety of vegetables, ornamentals, weeds, and other plants than other viruses [8]. In view of the fact that mixed infections involving some of these viruses are possible under the tropical environments of Nigeria, but there is dearth of information on such a phenomenon, the objective of this study was to examine the effects of single and mixed infection by CMeV and CMV on growth and yield parameters of six Cultivars of Soybean in Nigeria.

## Materials and methods

### Sourcing of seeds and propagation of soybean

The seeds of soybean cultivars; cv TGx 1844-18E, cv TGx 1019-2EN, cv TGx 1910-8F, cv TGx 1844-4E, cv TGx 1448-2E and cv TGx 1876-4E used in the experiment were collected from the International Institute of Tropical Agriculture (IITA), Ibadan, Nigeria. The seeds were sown in a 5 litre plastic buckets filled with sterilized sandy loam soil augmented with 5 g NPK fertilizer per litre soil at seedling at the rate of four seeds per pot and later thinned to two plants per pot. The pots were arranged in the screenhouse under ambient tropical temperature, lighting and humidity regimes between the months of December 2007 and April 2008.

### Source and propagation of inoculum and inoculation procedures

The CMeV and CMV isolates were extracted from infected leaves obtained from the stock of the Plant Pathology Laboratory at the International Institute for Tropical Agriculture (IITA). Infected leaf sample was macerated in phosphate buffer (pH 7.2) at the rate of 1 g/5 ml of buffer in pre-cooled mortar and pestle. The inoculation was done by mechanical transmission of virus through sap. The sap was applied on the surfaces of the oldest leaves previously dusted which carborundum. The sap was applied by rubbing the leaves gently with a cotton wool dipped in the sap. Inoculated plants were rinsed thereafter with water. Plants that were mock-inoculated with buffer only served as control. Inoculation was done two weeks after planting.

### Data collection and analysis

Data were collected at the time of infection as well as on weekly basis. Plant height and number of leaves were taken weekly over a period of 9 weeks after inoculation. Yield parameters such as number of pods, dry weight of pods (g) and dry weight of grain (g) were also taken. The pods were harvested, dried and weighed with the aid of an electronic balance. The pods were threshed manually and weighed, the treatment design was a factorial fitted into a randomized complete block design (RCBD). All data were subjected to analysis of variance (ANOVA) having regards for the factorial nature of the treatment design and the significant differences between them were determined at P < 0.05, using the new Duncan's Multiple Range test.

## Results

The six cultivars used for the experiment showed symptomatic response to infection by Cowpea mottle virus (CMeV) and Cucumber mosaic virus (CMV) under single and mixed infection situations but to seemingly different extent. TGx 1019-2EN and TGx 1448-2E were both susceptible to both mixed and single infection, TGx 1844-18E was susceptible to single infection, TGx 1910-8F was susceptible to mixed infection while TGx 1876-4E and TGx 1844-4E were mildly tolerant to both single and mixed infection.

**Figure 1:**
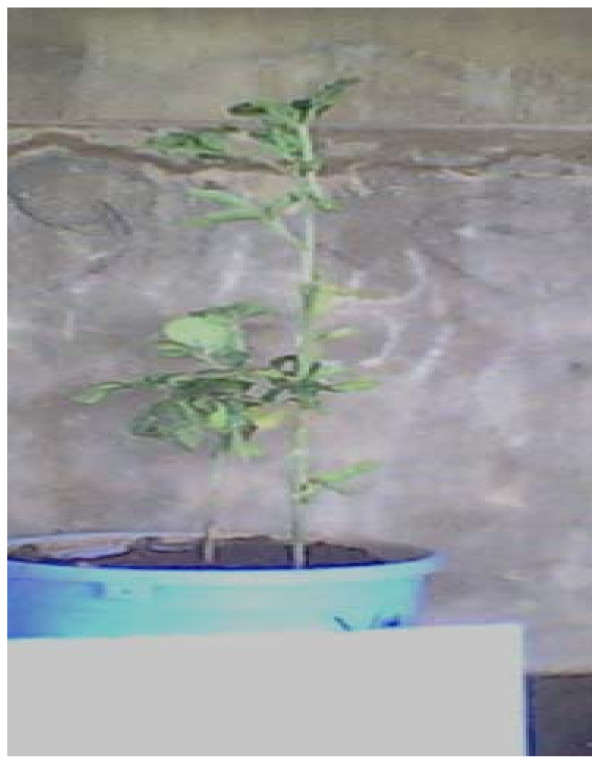
**Effect on growth**. Soybean cv TGx 1019-2EN under CMeV infection.

**Figure 2:**
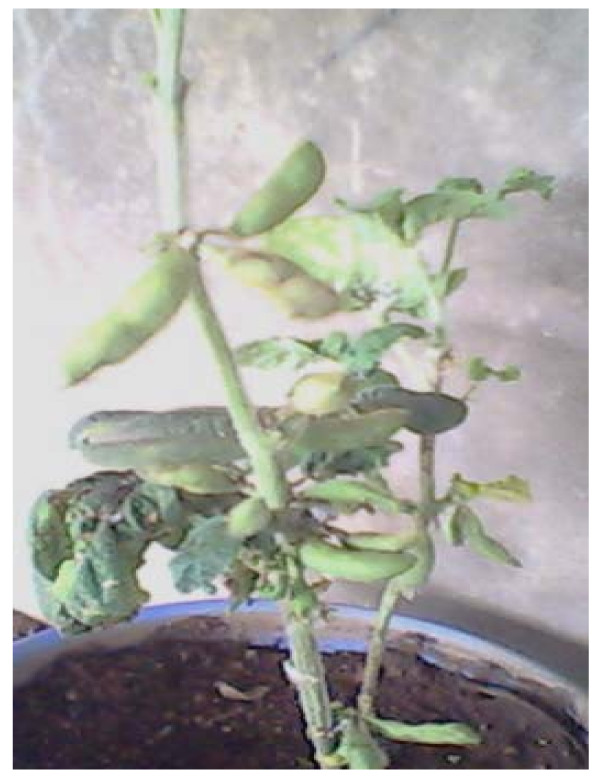
**Effects on yield attribute**. Fruiting pattern of diseased Soybean plant.

**Figure 3:**
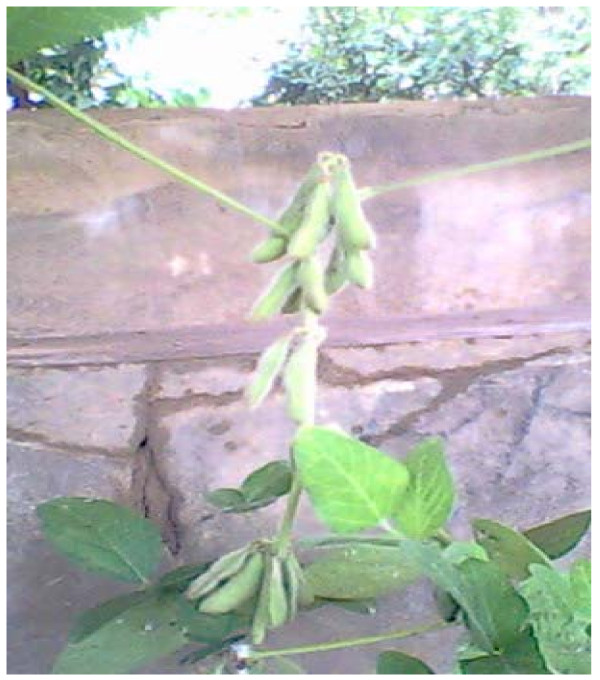
**Effects on yield attribute**. Fruiting patterns of healthy Soybean plant.

In susceptible cultivars infection with CMeV manifested as leaf mottling, which progressed to leaf wrinkling. Such leaves appeared relatively smaller in size than normal leaves. Generally, plants that were susceptible to infection with CMV alone manifested only mild mosaic symptoms while those plants that were susceptible to mixed infection with CMV and CMeV showed a combination of mosaic, necrosis and stunting as were also observed in severe CMeV infections (Figure 1). It was observed that CMeV induced striking symptoms even on the fruit setting and the fruits of severely infected plants. Figure 2 shows cv TGx 1019-2EN manifesting serious distortions on the fruit set. All mock inoculated control plants were free from the infections and had normal fruit set as shown in figure 3.

The main effect of variety and inoculation on growth parameters as at 7 weeks after inoculation as shown (Table 1), ranging from week 2 through week 7 after inoculation, viral inoculated plants were generally significantly shorter than the healthy control. However, there were no significant differences between inoculated plants until the 6^th ^and 7^th ^week. Even then, those inoculated singly with cowpea mottle virus and those inoculated with a mixture of cowpea mottle virus and cucumber mosaic virus were not significantly different. Consideration of the different treatment combinations showed that the soybean plants responded in various ways to the different inoculation regimes (Treatments). It is apparent however that those plants inoculated with CMeV were the most severely affected.

**Table 1:** Effect of variety and viral inoculation on the height of soybean at different times after inoculation

Weeks after inoculation								
**Variety**	**0**	**1**	**2**	**3**	**4**	**5**	**6**	**7**

TGx 1910 -- 8F	6.2 h	9.0 hi	13. 6 f	21.6 efg	31.2cde	40.5bcd	52.8a	55.6a
TGx 1448 -- 2E	10.0de	12.3de	16.3cde	22.6def	27.3ef	29.3g	34.3cde	36.9de
TGx1844-- 18E	14.8a	17.2a	20.6b	28.6bc	33.2bcd	42.2d	50.7a	54.4a
TGx 1844 -- 4E	9.1ef	11.5def	17.5c	24.1de	31.2cde	41.8bc	48.8a	50.7ab
TGx 1019--2EN	8.2fg	11.2efg	15.5cdef	22.6def	25.3f	29.3g	32.8def	36.3de
TGx 1876 -- 4E	5.6h	8.2i	13.4f	20.3fg	29.4cdef	46.9a	56.4a	64.2a
S. E	0.37	0.40	0.67	1.08	1.75	1.43	1.57	1.50
Viral Treatment								
CMeV	10.6a	12.9a	17.5b	24.1b	30.8b	36.5b	39.7c	40.8c
CMV	9.7b	12.1b	16.8b	24.5b	31.2b	38.1b	43.1b	43.7b
CMeV + CMV	9.3b	11.9b	16.8b	23.9b	30.4b	36.3b	39.8c	41.7c
Control	9.8b	12.4b	19.0a	28.0a	36.2a	41.2a	46.4a	54.4a
S. E	0.18	0.20	0.32	0.52	0.85	0.70	0.76	0.73

Means followed by the same letter(s) are not significantly different at P < 0.05 using the new Duncan's multiple range test

In the number of leaves, analysis of the main effect of inoculation (i.e. regardless of the variety involved) shows that there were generally significant differences between the viral inoculated soybean plants from week 2 through week 5 after inoculation. However, there were no significant differences between the control and CMV inoculated plants at 4^th^, 6^th ^and 7^th ^week after inoculation. There was no significant difference between all treatments including the control at week 8 as shown in Table 2.

**Table 2:** Effect of variety and viral inoculation on number of leaves of soybean at different times after inoculation

Weeks after inoculation										
**Variety**	**0**	**1**	**2**	**3**	**4**	**5**	**6**	**7**	**8**	**9**

TGx 1910 -- 8F	2.1f	3.2cd	4.5a	6.7a	8.0a	8.4b	9.2bc	8.8b	9.0b	8.6b
TGx 1448 -- 2E	2.3ef	3.0de	3.9cdef	5.4cdef	6.2efg	7.3c	7.8de	7.7cd	7.8cd	6.9cd
TGx1844-- 18E	2.0f	3.0de	3.9cdef	5.8bcde	6.3def	7.3c	8.2cd	7.8cd	7.5cde	6.7cd
TGx 1844 -- 4E	2.6cd	3.0de	3.9cdef	5.2def	6.7cde	8.1b	9.8b	8.2bc	8.6bc	7.4bcd
TGx1019--2EN	2.3ef	3.2cd	3.9cdef	5.2def	5.4g	5.7e	7.6def	7.3cd	7.5cde	7.1cd
TGx1876 -- 4E	2.0f	3.0de	4.0cdef	5.5bcdef	7.0bcd	9.8a	11.0a	12.0a	13.0a	10.9a
S.E	0.08	0.06	0.12	0.20	0.25	0.22	0.33	0.33	0.37	0.40
Viral Treatment										
										
CMeV	2.5a	3.0b	3.7c	5.4b	6.5b	7.2b	7.5b	7.5b	7.6a	6.8a
CMV	2.5a	3.1b	4.0b	5.6b	6.8ab	7.4b	8.3a	7.6ab	7.1a	6.0b
CMeV + CMV	2.4b	3.1b	3.9b	5.4b	6.5b	7.3b	7.5b	7.4b	7.4a	5.9b
Control	2.5ab	3.5a	4.5a	6.1a	7.1a	7.8a	8.1a	8.0a	7.6a	6.1b
S.E	0.04	0.03	0.06	0.10	0.12	0.10	0.16	0.16	0.18	0.19

Means followed by the same letter(s) are not significantly different at P < 0.05 using the new Duncan's multiple range test

### Effects on yield parameters

Table 3 shows the main effect of variety and inoculation on number of pods, weight of pods and weight of grain. As with the growth parameters, analysis of variance shows that the yield parameters in mock inoculated plants differed significantly from those in viral inoculated ones with mock inoculated plants having higher values compared to the viral inoculated ones.

**Table 3:** Effect of variety and viral inoculation on yield parameters

Variety	No. of pods	Dry weight of pods (g)	Dry weight of grains (g)
TGx 1910 -- 8F	19.8a	5.2a	3.9a
TGx 1448 -- 2E	14.7c	4.4abc	2.9abc
TGx1844-- 18E	8.3d	2.8de	1.7f
TGx 1844 -- 4E	19.3ab	5.0a	3.8a
TGx1019--2EN	16.8abc	5.0a	3.5a
TGx1876 -- 4E	15.4c	5.2a	3.4a
S.E	1.09	0.35	0.22
Viral Treatment			
			
CMeV	10.9c	3.1b	1.9b
CMV	13.0b	3.6b	2.1b
CMeV + CMV	12.3b	3.2b	1.9b
Control	18.1a	6.7a	5.3a
S.E	0.53	0.17	0.10

Means followed by the same letter(s) are not significantly different at P < 0.05 using the new Duncan's multiple range test

## Discussion

Recently, there has not been a record on the response of soybean cultivars to CMV in this part of the world. However, it has been reported that soybean in this part of the world is susceptible to cowpea mild mottle virus [4,1] but nothing had been said about mixed infection of the two viral diseases on soybean.

The experiment showed that all the cultivars of soybean used are susceptible to CMV, CMeV as well as a mixed infection with CMeV and CMV. The study showed that soybean cultivars; TGx 1844-18E and TGx 1019-2EN are highly susceptible to cowpea mottle virus as they expressed some symptoms showing deviation from the normal state of the plant physiology. Symptoms include stunting, mosaic pattern, mottling of the leaves and malformed leaves structures. Plants inoculated with CMV did not cause development of visible symptoms on some of the tested soybean cultivars. Smith [9] had also observed that CMV does not normally cause visible symptoms on Soybean. The soybean plants under mixed infection with CMV and CMeV showed symptoms similar to those manifested by plants under CMeV alone. This could be as a result of the effect of the CMeV in the combination. It was also an indication that the combination of the two viruses was not synergistic in the soybean cultivars.

## Abbreviations

CMeV: Cowpea mottle virus; CMV: Cucumber mosaic virus; TGx: Tropical glycine crossing.

## Competing interests

The authors declare that they have no competing interests.

## Authors' contributions

O participated in the design of the study, performed the inoculation, carried out data collection and drafted the manuscript. SO conceived the study, participated in its design, and coordination. TH participated in the design of the study and performed the statistical analysis. All authors read and approved the final manuscript.
